# Breast reconstruction and post-mastectomy radiation practice

**DOI:** 10.1186/1748-717X-8-45

**Published:** 2013-03-02

**Authors:** Susie A Chen, Crispin Hiley, Dana Nickleach, Janjira Petsuksiri, Fundagul Andic, Oliver Riesterer, Jeffrey M Switchenko, Mylin A Torres

**Affiliations:** 1 Department of Radiation Oncology, University of Texas Southwestern Medical Center, 5801 Forest Park Rd., Dallas, TX 75390-9183, USA; 2 The Institute of Cancer Research and The Royal Marsden National Health Service Foundation Trust, Sutton, Surrey, UK; 3 Biostatistics and Bioinformatics Shared Resource, Winship Cancer Institute, Emory University, 1365 Clifton Rd. NE, Building B, Atlanta, GA, USA; 4 Radiation Oncology Division, Radiology department, Faculty of Medicine Siriraj Hospital, Mahidol University, 2 Prannok Rd., Bangkoknoi, Bangkok, Thailand; 5 Department of Radiation Oncology, Cukurova University School of Medicine, Adana, Turkey; 6 Cukurova Universitesi Balcali Hastanesi, Radyasyon Onkolojisi, AD 01330, Saricam, Adana, Turkey; 7 Department of Radiation Oncology, University Hospital Zurich, Ramistrasse 100, 8091, Zurich, Switzerland; 8 Department of Radiation Oncology, Winship Cancer Institute, Emory University, 1365 Clifton Rd. NE, Rm 1307-A, Atlanta, GA 30322, USA

**Keywords:** Breast reconstruction, Post mastectomy radiation, Breast cancer, Survey

## Abstract

**Purpose:**

The goal of this study was to explore the perspectives and practice of radiation oncologists who treat breast cancer patients who have had breast reconstruction.

**Methods:**

In 2010, an original electronic survey was sent to all physician members of the American Society of Radiation Oncology, National Cancer Research Institute-Breast Cancer Studies Group in the United Kingdom, Thai Society of Therapeutic Radiology and Oncology, Swiss Society of Radiation Oncology, and Turkish Radiation Oncology Society. We identified factors associated with radiation oncologists who treat breast cancer patients with reconstruction performed prior to radiation and obtained information regarding radiation management of the breast reconstruction.

**Results:**

358 radiation oncologists responded, and 60% of the physicians were from the United States. While 64% of participants agree or strongly agree that breast image affects a woman’s quality of life during radiation, 57% feel that reconstruction challenges their ability to deliver effective breast radiation. Compared with other countries, treatment within the United States was associated with a high reconstruction rate (>/= 50% of mastectomy patients) prior to radiation (p < 0.05). Delayed-immediate reconstruction with a temporary tissue expander was more common in the United States than in other countries (52% vs. 23%, p = 0.01). Among physicians who treat patients with tissue expanders, the majority (60%) prefer a moderately inflated implant with 150-250 cc of fluid rather than a completely deflated (13%) or inflated expander (28%) during radiation. Among radiation oncologists who treat reconstructions, 49% never use bolus and 40% never boost a breast reconstruction. United States physicians were more likely than physicians from other countries to boost or bolus the reconstruction irrespective of the type of reconstruction seen in their clinic patients (p < 0.01).

**Conclusions:**

Great variation in practice is evident from our study of radiation treatment for breast cancer patients with reconstruction. Further research on the impact and delivery of radiation to a reconstructed breast may validate some of the observed practices, highlight the variability in treatment practice, and help create a treatment consensus.

## Introduction

Reconstruction following mastectomy for breast cancer enhances a woman’s body image and psychological well-being [1-3]. Although post-mastectomy radiation (PMRT) significantly improves survival in patients with high risk breast cancer [4-7], radiation to a reconstructed breast may affect breast symmetry, impair long term cosmetic outcome and potentially mitigate the life quality benefits of reconstruction [8-12]. Indeed, several studies have shown that PMRT may cause both short and long-term reconstruction complications resulting in infection, pain, poor wound healing, flap contraction, and implant extrusion [8,10,11,13,14]. Furthermore, previous research has suggested that reconstruction may negatively impact PMRT quality and delivery by increasing dose to the heart and lungs and by impairing chestwall and regional nodal coverage and overall outcome [15,16]. Given both the potential benefits and risks of reconstruction, there continues to be a significant amount of controversy regarding breast reconstruction and radiation treatment.

Prospective data supporting the most optimal reconstructive approach to women needing PMRT is lacking, and within this context, there are a variety of institutional preferences for the timing and type of reconstruction in breast cancer patients needing radiation. In addition, recent surgical advances have increased the number and complexity of reconstructive options available to women. Indeed, the impact of relatively novel reconstructive devices, such as internal magnetic metallic ports within temporary tissue expanders, on breast radiation is only beginning to be explored [17,18]. Nevertheless, mastectomy patients today have the following options: 1. No reconstruction, 2. Delayed reconstruction, 3. Delayed-immediate reconstruction with a temporary tissue expander, 4. Immediate autologous tissue flap reconstruction with or without an implant, and 5. Skin sparing mastectomy with or without preservation of the nipple and immediate implant placement.

Currently, there is no consensus on how to optimally incorporate reconstruction and PMRT into the overall breast cancer treatment plan. Moreover, there is no data documenting existing radiation practice and preferences based on clinic setting (academic vs. community), geographic locale, or the proportion of breast cancer patients seen and treated. Using an original electronic survey questionnaire, the goals of this study were: 1) to identify factors associated with radiation oncologists seeing a higher rate of breast reconstruction prior to radiation among their breast cancer patients and 2) to obtain information regarding radiation management of the breast reconstruction.

## Methods and materials

After obtaining Emory University Institutional Review Board approval, a cover letter explaining the study purpose and survey instrument were sent via electronic mail to physician members (including international members) of the American Society of Radiation Oncology (ASTRO), National Cancer Research Institute-Breast cancer Studies Group in the United Kingdom, Thai Society of Therapeutic Radiology and Oncology, Swiss Society of Radiation Oncology and Turkish Radiation Oncology Society. Between February and April 2010, the instrument was sent to potential English-speaking participants three times using Constant Contact Inc. Online Survey Service (Waltham, MA). Physicians in training and/or who do not administer radiation were asked to recuse themselves from participation. Eligible individuals wishing to participate provided informed consent before taking part in the survey. Those who responded to the survey could be distinguished from those who did not, and therefore, only physicians who did not reply were re-contacted for participation. Responses could not be linked to specific individuals.

The questionnaire used in this study collected demographic information including clinic location by country, practice type (academic vs. private practice), age, practitioner gender, and the proportion of patients treated with breast cancer following mastectomy with or without reconstruction as well as the type of reconstruction. The instrument also contained questions regarding physician perceptions of reconstruction during radiation and the ability to deliver effective radiation in this setting. For these items (See Additional file 1), participants were asked to rate their answers on a scale from 1 to 5 (1 = Strongly agree to 5 = Strongly Disagree). The other questions are detailed in Additional file 2. As tissue expanders with internal metallic ports are a relatively novel advancement in reconstruction, the last set of questions addressed specific considerations in planning PMRT in women with tissue expanders.

### Statistics

Descriptive statistics, including sample sizes and proportions, were generated for all variables. To make comparisons across reconstruction rate, we grouped physicians whose mastectomy patients have a reconstructed breast at least 50% of the time, as well as those whose patients have a reconstructed breast less than 50% of the time. We defined high volume breast cancer physicians as those where breast cancer represented ≥ 50% of their patient volume. Comparisons were also made across regions; for example, Developed Countries (United States of America (USA)/Canada/United Kingdom (UK)/Western Europe/Australia) vs. Developing Countries (Mexico/South America/Africa/Asia/Eastern Europe including Turkey/Middle East), and USA vs. Europe (Western), as the majority of respondents were from these two regions. Gender and age (<50 vs. ≥50) comparisons were also made. As all variables were categorical, comparisons across variables were analyzed using chi-squared tests, with Fisher’s Exact Test used for smaller sample sizes. Significance was assessed at the 0.05 level, and the analysis was performed in SAS Version 9.3.

## Results

### Demographic characteristics of participating physicians

The survey was sent to 4753 physicians. 358 (8%) agreed to participate. Each respondent’s clinic location by country is listed in Table 1. The majority of participating physicians were from the USA (60%), between 30 and 49 years of age (64%), and identified themselves as Caucasian (73%). More men (59%) than women (41%) responded to the survey. 46% of respondents noted at least half of their patients were individuals with breast cancer. Of note, significantly more female than male physicians had a high proportion of patients with breast cancer (≥50%) (61% vs. 36%, p < 0.001).

**Table 1:** Respondents’ radiation practice location by country

**Country**	**N = 358 (%)**
North America	
United States of America	182 (60)
Canada	12 (4)
Mexico	3 (1)
South America	
Argentina	2 (1)
Brazil	6 (2)
Chile	3 (1)
Europe	
Western Europe	
Austria	1 (0.3)
Belgium	2 (1)
France	3 (1)
Italy	3 (1)
Netherlands	7 (2)
Switzerland	2 (1)
United Kingdom	32 (11)
Eastern Europe	
Poland	1 (0.3)
Macedonia	1 (0.3)
Turkey	18 (6)
Asia	
China	1 (0.3)
India	2 (1)
Japan	1 (0.3)
Taiwan	2 (1)
Thailand	8 (3)
Middle East	
Egypt	1 (0.3)
Iran	1 (0.3)
Israel	1 (0.3)
Jordan	1 (0.3)
Saudi Arabia	1 (0.3)
Africa	
South Africa	3 (1)
Australia	1 (0.3)
New Zealand	1 (0.3)
Missing	56

Most participants (59%) stated that they work in an academic or academically-affiliated center. 54% of academically-affiliated vs. 46% non-academically-affiliated of physicians noted that they practice with six or more radiation oncologists (p = 0.40). More than half of the enrolled physicians (60%) serve an urban based patient population. The majority of radiation oncologists (90%) participate in a multidisciplinary breast tumor board, and 34% added that a reconstructive surgeon also attends their tumor board conference. Other relevant provider and radiation practice characteristics are shown in Table 2.

**Table 2:** Respondent practice characteristics

**Characteristic**	**N = 358 (%)**
% of Patients with Breast Cancer	
None	4 (1)
25%	152 (53)
50%	70 (24)
75%	45 (16)
100%	18 (6)
Missing	69
% of Breast Cancer Patients with Mastectomy	
None	6 (2)
25%	217 (77)
50%	49 (17)
75%	10 (3.5)
100%	1 (0.4)
Missing	75
% of Breast Cancer Patients with Reconstruction	
None	53 (19)
25%	154 (54)
50%	52 (18)
75%	24 (8)
100%	4 (1)
Missing	71

### Provider perceptions regarding reconstruction and radiation

Given 67% of respondents agree or strongly agree that women are concerned about their breast appearance during radiation, it was not surprising that 64% of physicians also agree or strongly agree that breast image during treatment affects a woman’s quality of life. However, while reconstruction improves body image, more than half (57%) of participants believe that reconstruction challenges their ability to deliver effective breast PMRT. Responses to these questions did not differ based on physician gender or age. In addition, high volume breast cancer physicians (50% or more of their patients have breast cancer) were not more likely than others to believe that reconstruction potentially effects the quality of their radiation (54% vs. 60%, p = 0.3).

### Mastectomy and reconstruction

Table 2 displays the proportion of breast cancer patients, post mastectomy breast cancer patients, and patients with reconstruction that participating physicians stated they see in their radiation clinics. 98% of participating radiation oncologists noted that at least one quarter of their breast cancer patients have been treated with mastectomy (as opposed to breast conserving surgery) when presenting for radiation. 82% of physicians stated that at least 25% of these mastectomy patients have also had breast reconstruction prior to radiation. A small, but not insignificant, number of respondents (19%) noted none of their mastectomy patients are reconstructed prior to PMRT. However, a larger proportion of radiation oncologists (27%) acknowledged that 50% or more of their patients are reconstructed before PMRT.

Potential factors associated with a higher mastectomy rate (≥50% of breast cancer patients) among breast cancer patients seen in the radiation clinic were then evaluated. Mastectomy was more commonly seen by participating radiation oncologists in developing rather than developed nations (58% vs. 12%, p < 0.001). Academically-affiliated centers were also more likely than private/community radiation practices to see a high proportion of post-mastectomy breast cancer patients (≥50% of breast cancer patients) (28% vs. 12%, p = 0.001) (See Table 3). Reconstructive surgery participation in multidisciplinary tumor board conference was associated with a low (<50% of breast cancer patients) rather than high mastectomy rate (14% vs. 25%, p = 0.02). There were no significant differences in mastectomy rate based on population served (urban vs. small city/rural).

**Table 3:** Mastectomy and reconstruction in academically-affiliated and non-academically affiliated radiation centers

**Characteristic**	**Academic N = 177 (%)**	**Non-Academic N = 125 (%)**
**% of Breast Cancer Patients with Mastectomy**		
None	2 (1.2)	4 (3.3)
25%	114 (70.8)	103 (84.4)
50%	36 (22.4)	13 (10.7)
75%	8 (5)	2 (1.6)
100%	1 (0.6)	0 (0)
Missing	16	3
**% of Breast Cancer Patients with Reconstruction**		
None	33 (20.0)	20 (16.4)
25%	89 (53.9)	65 (53.3)
50%	28 (17)	24 (19.7)
75%	14 (8.5)	10 (8.2)
100%	1 (0.6)	3 (2.5)
Missing	12	3

Factors potentially associated with a higher reconstruction rate were also examined. Significantly more radiation oncologists in developed countries treat a high proportion of patients with reconstruction (≥50% of mastectomy patients) than those in developing nations (32% vs. 10%, p = 0.001). American radiation oncologists were even more likely to treat reconstructions than their European counterparts (40% vs. 9%, p < 0.001). There were no significant differences in reconstruction rate based on population served (urban vs. small city/rural), practice type (academic vs. private practice), and reconstructive surgeon participation in tumor board (yes vs. no).

### Reconstruction by type

Among radiation oncologists who treat breast reconstructions (n = 234, See Table 4), the least common reconstruction treated was permanent implant with almost half (43%) of respondents stating that permanent implants are not placed in their patients before PMRT. One quarter of physicians also noted that autologous tissue flap reconstructions are not performed in their patients before PMRT. Placement of a temporary tissue expander appeared to be the most common form of reconstruction in women needing PMRT, as only 16% of radiation oncologists stated that tissue expander reconstruction is not performed in their patients before treatment.

**Table 4:** Reconstruction types seen by radiation oncologists who treat reconstructions

**Reconstruction Type**	**N = 234 (%)**
% of Patients with Permanent Implant	
None	101 (43.3)
25%	108 (46.4)
50%	16 (6.9)
75%	7 (3.0)
100%	1 (0.4)
Missing	1
% of Patients with Autologous Tissue Flaps	
None	59 (25.4)
25%	133 (57.3)
50%	26 (11.2)
75%	10 (4.3)
100%	4 (1.7)
Missing	2
% of Patients with Temporary Tissue Expanders	
None	38 (16.3)
25%	98 (42.1)
50%	37 (15.9)
75%	42 (18.0)
100%	18 (7.7)
Missing	1

The country where the provider practiced was significantly associated with the type of reconstruction performed prior to PMRT. More American than European radiation oncologists treated temporary tissue expanders in 50% or more of their reconstructed patients (52% vs. 14%, p < 0.001), whereas more European than American physicians treated autologous tissue flaps in this same proportion of patients (36% vs. 14%, p = 0.002). Radiation oncologists who see a relatively high volume of breast cancer patients (50% or more of their patients) were not more likely to see a specific type of reconstruction in their patients.

### Radiation treatment

**Figure 1:**
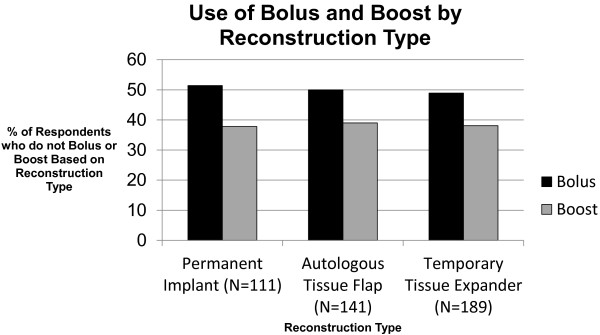
Use of bolus and boost by reconstruction type.

Among the subset of respondents who treat reconstructions with radiation (n = 234), almost half never use bolus (49%) or boost (40%) the reconstructed breast. The proportion of radiation oncologists who never treat the reconstruction with bolus or boost was similar irrespective of the type of reconstruction typically seen (≥25% of reconstructed breasts) by participating physicians in their clinics (Figure 1). High volume breast cancer radiation oncologists do not use bolus (54% vs. 49%, p = 0.42) or prescribe boost dose to the reconstruction (64% vs. 57%, p = 0.31) more frequently than radiation oncologists who treat a lower volume of breast cancer patients. The age of the participating physicians was also not associated with whether or not a patient with reconstruction was treated with bolus. Physicians at least 50 years of age or older, however, were more likely to prescribe boost dose than younger physicians to the reconstruction (69% vs. 55%, p = 0.04). There were no differences in the use of bolus or boost to the reconstruction based on the gender of the provider.

**Figure 2:**
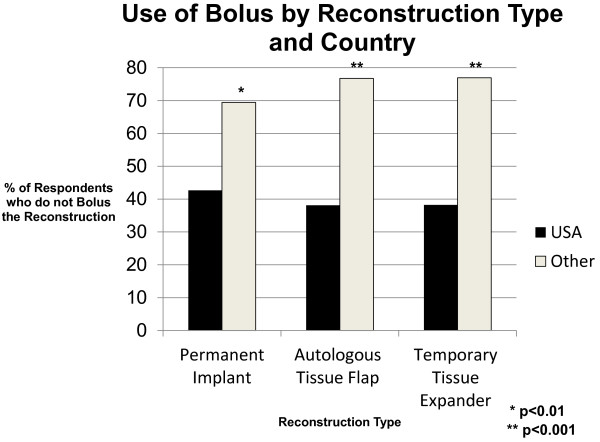
Use of bolus by reconstruction type and country.

**Figure 3:**
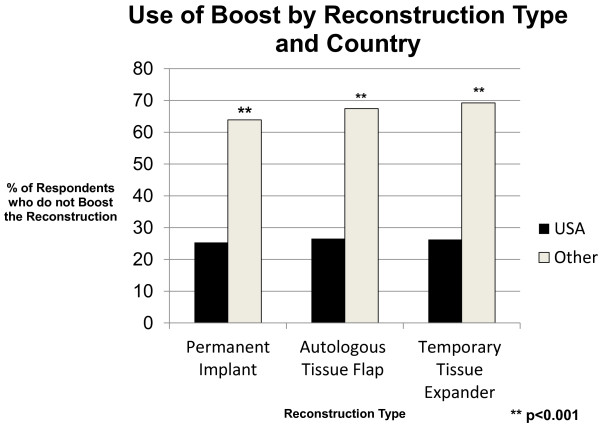
Use of boost by reconstruction type and country.

Based on the varying rates of reconstruction and reconstruction types seen by radiation oncologists worlwide, radiation treatment of the reconstruction was examined based on geographic locale and only among those providers who stated that they see reconstructed breast cancer patients in their radiation centers. Compared with physicians from other countries (n = 81), American radiation oncologists (n = 152) treat more reconstructions with bolus (62% vs. 24%, p < 0.001) and more frequently prescribe a boost dose to the reconstruction (72% vs. 29%, p < 0.001) irrespective of reconstruction type (See Figures 2 and 3).

In comparing physicians from the USA (n = 152) and Europe (n = 36) specifically, more American than European radiation oncologists use bolus (62% vs. 29%, p = 0.002) and prescribe a boost dose (72% vs. 17%, p < 0.001) to the reconstruction. However, when reconstruction type was taken into account in the analysis, American radiation oncologists were not more likely than European physicians to administer bolus to a reconstructed breast with a permanent implant (58% vs. 40%, p = 0.20), but they were more likely to give the permanent implant a boost dose (76% vs. 20%, p < 0.001). In addition, Americans were still more likely to bolus and boost the reconstruction than Europeans if the reconstruction was an autologous tissue flap (63% USA vs. 26% European physicians bolus; 74% USA vs. 16% European physicians boost) or temporary tissue expander (62% USA vs. 26% European physicians bolus; 74% USA vs. 17% European physicians boost) (p < 0.01 for all comparisons).

### Tissue expanders

In the cohort of radiation oncologists who treat patients with temporary tissue expander implants (n = 211), significantly more physicians only treat patients with internal rather than external ports (62% vs. 6%, p < 0.001) and significantly more prefer an internal rather than external location for access to the expander (85% vs. 15%, p < 0.001). However, only 35% agree or strongly agree that the location of the port affects radiation dose distribution and/or challenges radiation treatment planning.

A small number of physicians (13%) prefer a completely deflated implant during radiation, while 28% prefer a completely inflated implant during PMRT. The vast majority of radiation oncologists (60%) prefer 150-250 cc of fluid within the expander to facilitate PMRT planning. Two thirds (66%) of physicians agreed or strongly agreed that the volume of fluid within the implant affects radiation dose distribution and can make radiation treatment planning challenging, but only 47% of radiation oncologists will ask reconstructive surgeons to adjust the expander volume to 150-250 cc. Respondents who stated that plastic surgeons attend their tumor board were more likely to request tissue expander volume adjustments to facilitate PMRT planning (45% vs. 32%, p = 0.07).

The most common reason for requesting a moderately sized expander was to minimize dose to critical structures including the heart and lungs (39%). Fewer radiation oncologists (14%) will ask for adjustments in implant volume for the purposes of treating the internal mammary lymph nodes. 91% of physicians stated that the laterality (right vs. left) of the breast cancer did not affect their decision to request a moderately sized expander. Of note, in patients with bilaterally reconstructed breasts with temporary tissue expanders, 58% of physicians will request that the implant volume be adjusted to 150–250 cc in the contralateral non-cancerous breast to minimize radiation dose to this unaffected breast. Treating a large proportion of breast cancer patients (≥ 50% of patients) was not associated with a preference for an internal or external port, the approach to a tissue expander, or the reasons for requesting a moderately sized implant. In addition, female and male providers did not have significantly different responses to these questions.

## Discussion

Our study indicates great variation in both the rate and type of reconstruction in breast cancer patients seen by radiation oncologists. However, radiation oncologists from developed nations see a larger proportion of their breast cancer patients treated with reconstruction before radiation than those from developing countries. Moreover, American respondents see more patients with breast reconstruction than European physicians although the relative number of participating European physicians was small. While respondents from academic-affiliated centers appeared to see more mastectomy patients than non-academically affiliated centers, there was no significant difference between the two groups in the proportion of breast cancer patients who had undergone breast reconstruction. Admittedly, the higher rate of mastectomy patients seen among participating academic physicians is likely due to differences in referral patterns with a higher proportion of patients with advanced disease needing mastectomy being seen and treated in academically-affiliated centers.

American radiation oncologists more frequently encountered patients with breast reconstruction in their clinics and were more likely than others to use bolus and/or boost the reconstruction. Placement of a temporary tissue expander was the most common form of reconstruction seen by respondents, and Americans were more likely than others to see this reconstruction type. As providers who treat tissue expanders may be more apt to bolus or boost a tissue expander than an autologous tissue flap, analysis was performed taking into account reconstruction type. There was no difference in radiation management based on reconstruction type. Americans were still more likely than others to boost or bolus any form of reconstruction (permanent implant, autologous tissue flap, or temporary tissue expander). Moreover, American physicians were more likely than European physicians to boost or bolus an autologous tissue flap or temporary tissue expander. This finding is consistent with the observation that American ph*y*sicians are also more likely than Europeans to use bolus in post-mastectomy patients without reconstruction [19].

Nevertheless, our data indicate that less than half of American physicians, (albeit significantly more than European physicians), will bolus and/or boost the chestwall when a reconstruction is present. Randomized trials of boost and bolus in the post-mastectomy setting are lacking, but current practice suggests that reconstructed breast cancer patients are often treated without bolus and to a lower total dose than mastectomy-only patients due to concerns over complications and cosmetic outcome. Common indications for using bolus and/or boost (e.g. close or positive margins, skin involvement by the tumor, and tumor size), however, presumably exist in both reconstructed and non-reconstructed patients. Future prospective studies examining long term outcomes are needed to determine if bolus and/or boost provide a recurrence free benefit and evaluate whether the observed current radiation practice in women with reconstruction is potentially impairing long term cancer control.

Although physicians agree that reconstruction may improve the life quality of a patient during radiation, most radiation oncologists in this study feel that reconstruction also challenges their ability to deliver effective treatment. Tissue expanders may provide some compromise although patients are inconvenienced by a second major surgical procedure for permanent implant placement and potential complications [8,10,17]. Tissue expander reconstruction, however, allows a woman to have a breast mound whose volume may be adjusted to minimize its impact on radiation quality. Indeed, a number of providers feel that decreasing the size of the expander to 150 to 250 cc from a fully expanded implant improves radiation treatment and delivery by decreasing dose to the heart and lung. However, less than half of radiation providers will ask reconstructive surgeons to adjust the volume within an expander suggesting that either institutional protocols regarding expander volume may already be in place or that communication between radiation oncologists and reconstructive surgeons could be improved. Others have also noted that earlier inclusion of radiation oncologists in the overall treatment planning process of the patient may influence patient decisions regarding surgical treatment and could possibly influence a patient’s choice to undergo reconstruction [20]. Indeed, a multidisciplinary tumor board may function as a venue for improved communication between providers, and our data seems to support this notion, as respondents who stated that reconstructive surgeons attend their tumor board were more likely to ask for tissue expander volumes to be adjusted to facilitate radiation treatment planning.

Limitations of this study include a low overall response rate and the possibility that information reported by the participating radiation oncologists does not truly represent their practice. Nevertheless, surveys have been used previously to more broadly understand the scope of practice within various medical fields including radiation oncology [20-22]. This questionnaire was not formally validated and was only sent three times to potential participants, which may have contributed to the relatively small number of participating physicians. It is also likely that physicians who do not treat breast cancer were also less likely to respond to the survey limiting the potential number of respondents. As most of the participants were members of ASTRO and the instrument was written only in English, our findings emphasize the perspectives of English speaking physicians who are mostly American. Furthermore, willing participants may have had a greater interest in radiation and reconstruction due to frequent experience with reconstruction in their patients. Therefore, the study results could be biased to reflect the views of physicians frequently encountering reconstruction although an effort was made to assess respondents based on the rate of reconstruction seen among breast cancer patients in their radiation center. Results regarding treatment of the reconstruction were purposely limited to radiation oncologists who see patients with breast reconstruction to determine the current practice of those physicians, as data regarding radiation management of breast reconstruction is lacking. The study was admittedly limited to radiation society membership lists and e-mail addresses to which we had access, resulting in poor response rates from physicians located in South America, Asia, and Africa.

This small study, however, represents the first attempt to document and establish a baseline of practice regarding reconstruction and the radiation approach to reconstruction in physicians from multiple countries. Prospective research on the impact and delivery of radiation to a reconstructed breast is needed to validate the observed practice and aid in creating a generalized treatment consensus.

## Conclusions

Collectively, findings from this study suggest that there continue to be a variety of approaches to radiation treatment of a reconstructed breast following mastectomy. Among the respondents, the most common form of reconstruction in patients presenting for radiation was placement of a temporary tissue expander. However, when treating a reconstruction, many radiation oncologists will not use bolus or boost the reconstruction regardless of reconstruction type.

## Competing interests

The authors declare that they have no competing interests.

## Authors’ contributions

SC made substantial contributions in concept and design of the study. She was primarily involved in acquisition of data, analysis and interpretation of data. She has been involved in drafting the manuscript and gave final approval of the version to be published. CH made substantial contributions in concept and design of the study. He helped identify potential UK radiation physicians for participation in the study. He was involved in analysis and interpretation of data. He was involved in drafting and revising the manuscript for intellectual content and gave final approval of the version to be published. DN made substantial contributions in design of the study and was heavily involved in the analysis and interpretation of data. She was involved in drafting and revising the manuscript for intellectual content and gave final approval of the version to be published. JP made substantial contributions in concept and design of the study and identifying potential radiation oncologists in Thailand for participation in the study. She was involved in data acquisition and in drafting and revising the manuscript for intellectual content and gave final approval of the version to be published. FA made substantial contributions in concept and design of the study and identifying potential radiation oncologists in Turkey for participation in the study. She was involved in data acquisition and in drafting and revising the manuscript for intellectual content and gave final approval of the version to be published. OR made substantial contributions in concept and design of the study and identifying potential radiation oncologists in Switzerland for participation in the study. He was involved in data acquisition and in drafting and revising the manuscript for intellectual content and gave final approval of the version to be published. JS made substantial contributions in design of the study and was heavily involved in the analysis and interpretation of data. He was involved in drafting and revising the manuscript for intellectual content and gave final approval of the version to be published. MT made substantial contributions in concept and design of the study. She was involved in acquisition of data, analysis and interpretation of data. She helped identify potential radiation oncologists within the United States for participation. She has made substantial contributions to drafting and revising the manuscript for intellectual content and gave final approval of the version to be published. All authors read and approved the final manuscript.

## Authors’ information

MT is an Assistant Professor of Radiation Oncology at Emory University where she specializes in breast cancer. She is also a Radiation Therapy Oncology Group Breast Committee Member. Through discussions with academic and non-academic radiation oncologists, it is clear that breast reconstruction is becoming more prevalent, particularly in the United States, and there is no consensus on how to treat breast reconstructions with radiation. This study was designed to determine current practice among both academic and non-academic radiation oncologists from a variety of countries.

Meeting Presentation: 52^nd^ Annual American Society for Radiation Oncology, November 2010, San Diego, CA

## Supplementary Material

Additional file 1 Perception questions. Click here for file

Additional file 2 Additional survey questions. Click here for file
