# Supplementary material for: Breast reconstruction and post-mastectomy radiation practice
Source: Radiat Oncol. 2013 Mar 2;8:45. doi: 10.1186/1748-717X-8-45 (PMC3599934; doi:10.1186/1748-717X-8-45)
Supplement: Additional file 1 — Perception questions. [file 1748-717X-8-45-S1.docx]

**Figure 1. Perception Questions**

1. **Breast image during radiation affects a woman’s quality of life.**
   1. **Strongly Agree**
   2. **Agree**
   3. **Neutral**
   4. **Disagree**
   5. **Strongly Disagree**
2. **Female patients are concerned about their breast appearance during radiation.**
   1. **Strongly Agree**
   2. **Agree**
   3. **Neutral**
   4. **Disagree**
   5. **Strongly Disagree**
3. **Reconstruction challenges your ability to deliver effective breast radiotherapy care**
   1. **Strongly Agree**
   2. **Agree**
   3. **Neutral**
   4. **Disagree**
   5. **Strongly Disagree**
