# Supplementary material for: Breast reconstruction and post-mastectomy radiation practice
Source: Radiat Oncol. 2013 Mar 2;8:45. doi: 10.1186/1748-717X-8-45 (PMC3599934; doi:10.1186/1748-717X-8-45)
Supplement: Additional file 2 — Additional survey questions. [file 1748-717X-8-45-S2.docx]

Additional Survey Questions:

1. What percent of the patients you treat with radiation have breast cancer?

a) None

b) 25%

c) 50%

d) 75%

e) 100%

2. What percent of your breast cancer patients have undergone mastectomy prior to radiation?

a) None

b) 25%

c) 50%

d) 75%

e) 100%

1. Among your post-mastectomy patients, what percent of patients have a reconstructed breast (autologous tissue flap, permanent implant or temporary implant) prior to and during radiation?

a) None

b) 25%

c) 50%

d) 75%

e) 100%

- - 1. What percent of reconstructed breasts you treat have permanent implants placed before radiation?

a) None

b) 25%

c) 50%

d) 75%

e) 100%

- - 1. What percent of your reconstructed patients have autologous tissue flaps (i.e. transverse rectus abdominis myocutaneous (TRAM) flap, latissimus dorsi flap) placed before radiation?

a) None

b) 25%

c) 50%

d) 75%

e) 100%

- - 1. What percent of your patients with reconstructed breasts have temporary tissue expanders placed before radiation?

a) None

b) 25%

c) 50%

d) 75%

e) 100%

1. Approximately, what percent of reconstructed breasts (autologous tissue flap, permanent implant or temporary implant), do you treat with bolus?
   1. None
   2. 25%
   3. 50%
   4. 75%
   5. 100%
2. Approximately, what percent of reconstructed breasts (autologous tissue flap, permanent implant or temporary implant) do you give a boost dose?
   1. None
   2. 25%
   3. 50%
   4. 75%
   5. 100%
3. In your patients with temporary tissue expanders, do you prefer the expander to be completely deflated, fully expanded, or of moderate size (e.g. 150-250cc) during radiation?
   1. Completely deflated
   2. Fully expanded
   3. Moderate size
4. In patients with temporary tissue expanders, the volume contained within the implant affects the radiation dose distribution or makes radiation treatment planning challenging.
   1. Strongly Agree
   2. Agree
   3. Neutral
   4. Disagree
   5. Strongly Disagree
5. In your patients with temporary tissue expanders, approximately how often do they have internal magnetic ports?

a) None

b) 25%

c) 50%

d) 75%

e) 100%

1. In your patients with temporary tissue expanders, approximately how often do they have external ports?

a) None

b) 25%

c) 50%

d) 75%

e) 100%

1. In your patients with temporary tissue expanders, do you prefer internal or external ports in temporary tissue expanders?
   1. Internal Ports
   2. External Ports
2. In patients with temporary tissue expanders, the location of the port affects radiation dose distribution and/or challenges radiation treatment planning.
   1. Strongly Agree
   2. Agree
   3. Neutral
   4. Disagree
   5. Strongly Disagree
3. In your patients with temporary tissue expanders, how often do you ask the plastic surgeon to insert a small amount of saline (i.e. 150-200cc) rather than full expansion or complete deflation for radiation treatment purposes?
   1. Never
   2. 25% of the time
   3. 50% of the time
   4. 75% of the time
   5. 100% of the time
4. If you ask for a “moderate implant volume” in women with temporary tissue expanders, does this occur more often when you are treating right- or left-sided reconstructed breast?
   1. Right Breast
   2. Left Breast
   3. No Difference
5. If you ask for a “moderate implant volume” in women with temporary tissue expanders, does this occur more often when you are targeting the internal mammary nodes with radiation?
   1. Yes
   2. No
6. If you ask for a “moderate implant volume” in women with temporary tissue expanders, does this occur more often when trying to decrease the dose to organs (i.e. heart or lung) at risk?
   1. Yes
   2. No
7. In bilateral reconstructed breasts with temporary tissue expanders, do you ask for a moderate implant volume (150-250ccs) in the contralateral unaffected breast to decrease radiation dose to the contralateral breast and optimize dose coverage of the affected breast?
   1. Yes
   2. No
